# A case report of patient with severe acute cholangitis with tigecycline treatment causing coagulopathy and hypofibrinogenemia

**DOI:** 10.1097/MD.0000000000009124

**Published:** 2017-12-08

**Authors:** Xiaoqin Wu, Ping Zhao, Liang Dong, Xiuhong Zhang

**Affiliations:** a Department of Pharmacy, Ruijin Hospital Suzhou Branch Affiliated to Shanghai Jiaotong University School of Medicine; b Department of Pharmacy, Jiangsu Shengze Hospital, Suzhou; c Department of Critical Care Medicine; d Department of Pharmacy, Wuxi People's Hospital Affiliated to Nanjing Medical University, Wuxi, PR China.

**Keywords:** adverse reaction, coagulopathy, hypofibrinogenemia, tigecycline

## Abstract

**Rationale:**

Tigecycline is the first member of the glycylcycline family. There are rarely reports of tigecycline causing coagulopathy and hypofibrinogenemia until now. We report a case on tigecycline-associated coagulopathy and hypofibrinogenemia and discuss the characteristics of the adverse reaction.

**Patient concerns:**

A 47-year-old male patient with severe acute cholangitis who developed sepsis was treated with a high dosage (100 mg twice daily) of tigecycline. He experienced coagulopathy and hypofibrinogenemia as substantiated by increased levels of prolonged prothrombin time (PT), the international normalized ratio (INR) and activated partial thromboplastin time (APTT), and in particular, the fibrinogen (FIB) levels obviously decreased.

**Diagnoses:**

Coagulopathy and hypofibrinogenemia.

**Interventions:**

We discontinued tigecycline and gave the patient several blood products to prevent spontaneous bleeding.

**Outcomes:**

The adverse reaction disappeared after the withdrawal of tigecycline. After 30 days of hospitalization, the patient discharged with symptom free.

**Lessons:**

We suggest that coagulation parameters should be closely monitored in patients treated with tigecycline, specifically in patients who may be renal insufficiency, female or use the high-dose.

## 1 Introduction

Tigecycline, the first member of the glycylcycline family, was launched on the market in June 2005 by the United States Food and Drugs Administration (FDA), and it was listed in December 2011 in China. It is indicated in patients 18 years of age and older for: complicated skin and skin structure infections, complicated intra-abdominal infections, and community-acquired bacterial pneumonia. As tigecycline has a broad spectrum of gram-positive and gram-negative aerobic and anaerobic bacteria, and it is especially useful against extensively drug-resistant (XDR) bacteria, so it has been widely used. Then, tigecycline-associated adverse reactions are increasing. Kadoyama et al^[1]^ summarized a total of 248 tigecycline-related adverse reactions, the most common being nausea, vomiting, elevated levels of alanine aminotransferase, bilirubin, alkaline phosphatase, and aspartate aminotransferase, and hepatic dysfunction. However, there are rarely reports of tigecycline causing coagulopathy and hypofibrinogenemia until now. Here, we report a case of 1 patient with severe acute cholangitis and septic shock who experienced coagulopathy and hypofibrinogenemia that were caused by tigecycline. We discuss the characteristics of tigecycline-associated coagulopathy and hypofibrinogenemia and suggest clinicians should be aware of the potential for coagulopathy and hypofibrinogenemia when using tigecycline, specifically in patients who may be renal insufficiency, female or use the high-dose.

## 2 Case presentation

A 47-year-old male patient was admitted to the intensive care unit (ICU) due to severe acute cholangitis on May 30, 2016 with a history of type II diabetes, chronic pancreatitis, and pancreatic duct stones. The patient did not have any family history of hereditary bleeding disorder or underlying hepatic disease. In 1995, the patient underwent a cholecystectomy surgery, and he was then diagnosed with choledoco duodenal fistula (CDF). The patient received 1 g of imipenem–cilastatin intravenously every 8 hours for a bacterial infection and other symptomatic treatments.

On the 3rd day of hospitalization, the patient's infection exacerbated with an elevated white blood cell count (WBC) of 19.1 × 10^3^/μL, C-reactive protein (CRP) of 68 mg/L and a high temperature of 40°C. Then, hypoxemia declined to 84%, the heart rate increased, and dyspnea followed. Thus, severe acute pulmonary edema and acute respiratory distress syndrome (ARDS) caused by serious secondary biliary infection were considered. In addition, blood culture revealed methicillin-resistant *Staphylococcus warneri* (MRSW) which was sensitive to tigecycline and bile culture was positive for Candida albicans. Therefore, continuous veno-venous hemofiltrationhead (CVVH) was started to clear the inflammatory mediators, and tigecycline (initiated with a 100-mg loading dose, followed by 100 mg twice daily) and caspofungin were added. The coagulation parameters were within the normal range before treatment.

On the 4th day of hospitalization, the patient's infection improved with a dropped temperature (37.2°C), WBC (9.2 × 10^3^/μL), and CRP (58 mg/L). Nevertheless, increased levels of prolonged prothrombin time (PT), international normalized ratio (INR), and activated partial thromboplastin time (APTT) were observed; furthermore, the fibrinogen (FIB) levels obviously decreased.

**Figure 1:**
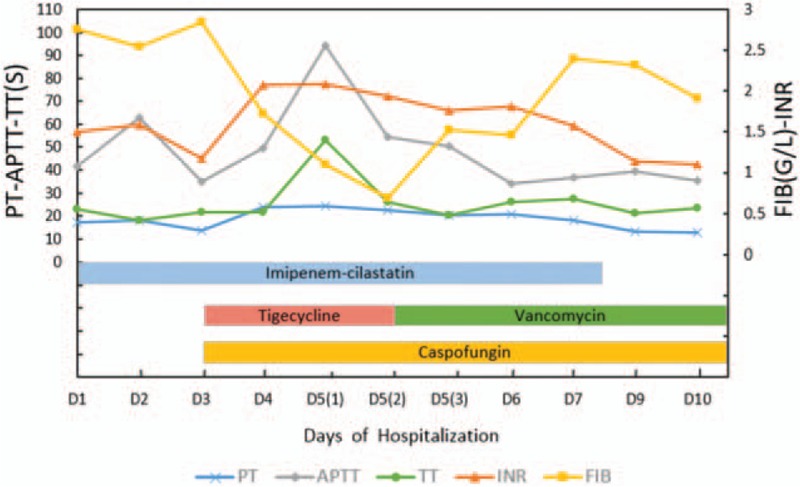
The patient's coagulation parameters relative to the time courses of his antimicrobial treatments during hospitalization. The patient was started empirically on imipenem–cilastatin for bacterial therapy on the 1st day of hospitalization. On day 3, the patient's infection exacerbated, tigecycline and caspofungin were added. However, the coagulation parameters were within the normal range on that day and began to be abnormal from the next day. A progressive worsening of coagulation parameters (measured twice**)** was noted on day 5. Then, we discontinued tigecycline and switched to vancomycin, in addition, several blood products were given to the patient. The patient's coagulation parameters improved after discontinuation of tigecycline and rescored to baseline levels on the 10th day of hospitalization. APTT (gray line) = activated partial thromboplastin time, FIB (yellow line) =  fibrinogen, INR (orange line) = international normalized ratio, PT (blue line) = prothrombin time, TT (green line) = thrombin time.

After 2 days of tigecycline treatment, the infection improved significantly, but a progressive worsening of coagulation parameters was noted. The PT increased from 13.6 to 24.3 seconds, the INR increased from 1.18 to 2.09, the APTT increased from 35.1 to 94.5 seconds, the thrombin time (TT) increased from 18.2 to 53.0 seconds, and the FIB levels decreased from 2.84 to 0.69 g/L (Fig. 1). After ruling out other causes, tigecycline was suspected as the most likely offending agent; thus, we discontinued tigecycline and switched to vancomycin. In addition, the patient received several blood products (cryoprecipitate infusion, a prothrombin complex, and fibrinogen infusion) to prevent spontaneous bleeding, although the patient had no clinical evidence of bleeding.

The patient's coagulation parameters rescored to baseline levels 5 days (Fig. 1) after discontinuation of tigecycline. After 16 days of hospitalization, the patient was in good condition and discharged from the ICU. The patient's infection was gradually controlled by effective antimicrobial treatment when he was in the general ward. He discharged with symptom free on June 28, 2016.

## 3 Discussion

Tigecycline has a broad spectrum of action, especially against multidrug-resistant (MDR) organisms, such as methicillin-resistant *Staphylococcus aureus* (MRSA), vancomycin-resistant enterococci (VRE), extended spectrum β-lactamase (ESBL)-producing gram-negative bacteria, and *Acinetobacter baumannii*. Since then, it has been widely used.^[2]^ Tigecycline requires intravenous administration with a loading dose of 100 mg followed by 50 mg every 12 hours. No dose adjustment is required for patients with severe renal impairment.^[3]^ However, the dosage should be reduced to 25 mg every 12 hours with severe hepatic dysfunction (Child Pugh C).^[4]^

The instructions for tigecycline prompt adverse reactions in the hemic and lymphatic system: partial thromboplastin time (APTT), prolonged prothrombin time (PT), eosinophilia, increased international normalized ratio (INR), and thrombocytopenia. On the additional literature review, there are few but severe cases regarding tigecycline-associated coagulopathy and hypofibrinogenemia that have been reported (Table 1). In our case, the patient received antimicrobial therapy with a high dose of tigecycline (100 mg twice daily), which was used off-label. Indeed, it has been found that a high dose of tigecycline treatment in ICU patients results in a decrease in fibrinogen concentration and an increase in INR and APTT values.^[8]^ Otherwise, studies have noted that systemic inflammation will invariably lead to activation of the coagulation system, and vice versa, components of the coagulation system may markedly modulate the inflammatory response.^[9]^ Thus, it is possible that sepsis contributed to the development of coagulation failure. Nevertheless, in the calculation of the Naranjo Adverse Drug Reaction Probability Scale,^[10]^ points were allocated for the presence of previous conclusive reports on tigecycline-associated coagulopathy and hypofibrinogenemia (1 point), the occurrence of coagulopathy after administration of the tigecycline (2 points), improvement of coagulopathy when tigecycline was discontinued and a cryoprecipitate and others were administered (1 point), more severe coagulopathy when the dose was increased (1 points), and confirmation by objective evidence (1 point). One point was subtracted from the score due to the presence of alternative causes of coagulopathy (sepsis leading to activation of the coagulation system). This resulted in a total of 5 points, which categorizes this adverse drug reaction as probable (Table 2).

**Table 1:**
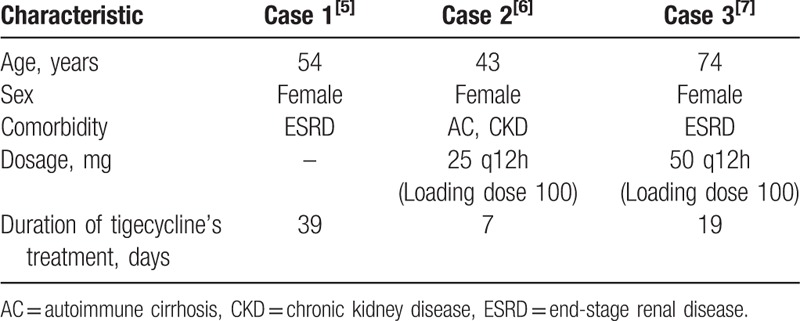
Cases regarding tigecycline-associated coagulopathy and hypofibrinogenemia that have been reported.

**Table 2:**
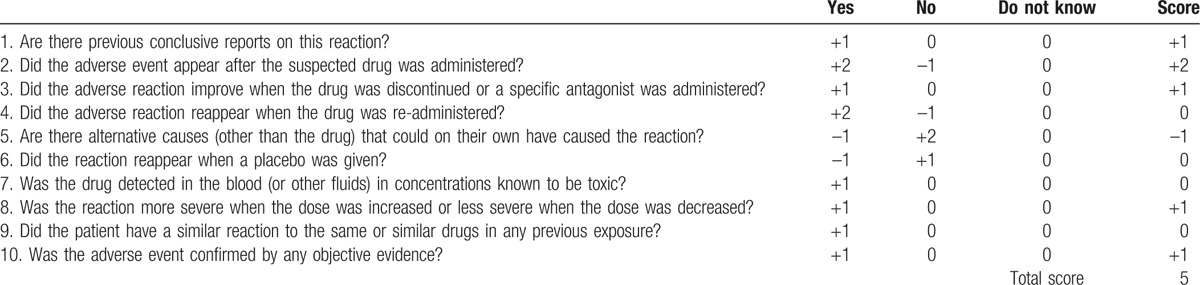
Adverse drug reaction probability scale.

The main mechanism by which tigecycline induces coagulopathy and hypofibrinogenemia is unclear. Tigecycline, which is structurally similar to tetracycline class antibiotics, can influence coagulation via either an effect on the vitamin-K-producing flora of the gut or a direct effect on the coagulation cascade.^[8]^ The instructions for tigecycline prompt adverse reactions that prolonged APTT, PT, and eosinophilia and increased INR and thrombocytopenia. However, a tigecycline-associated decrease in fibrinogen levels is a new serious adverse reaction. Zhang et al^[11]^ reported that the FIB levels decreased severely in the higher-dose group, and there was no difference in the decrease in FIB levels or the FIB level recovery by age. Whether the decreased concentration of fibrinogen found was due to increased consumption or to impaired synthesis is questionable.^[8]^ Furthermore, a study showed that the underlying mechanisms remain to be further elucidated with emphasis on the potential role of genetic susceptibility.^[7]^ Based on the reports of tigecycline-associated coagulation disorder,^[5–7]^ we found that gender (female), renal insufficiency and high-dose use may be risk factors for the adverse reactions.

## 4 Conclusion

In conclusion, tigecycline may induce prolonged coagulopathy with PT, APTT, and reduced FIB. In our case, the patient was treated with a high dose of tigecycline (100 mg twice daily), and coagulopathy was found on the next day. The coagulation parameters normalized within 5 days after tigecycline discontinuation. Thus, to ensure the safety of drug treatment, using the recommended dose is necessary. Consequently, we suggest that coagulation parameters should be closely monitored in patients treated with tigecycline, specifically in patients who may be renal insufficiency, female or use the high dose.

## Acknowledgments

The authors would like to express their appreciation to all of the medical staff in the intensive care unit of Wuxi People's Hospital Affiliated to Nanjing Medical University.
